# Narrow Pore Crossing of Active Particles under Stochastic Resetting

**DOI:** 10.3390/e25020271

**Published:** 2023-02-01

**Authors:** Weitao Zhang, Yunyun Li, Fabio Marchesoni, Vyacheslav R. Misko, Pulak K. Ghosh

**Affiliations:** 1 Center for Phononics and Thermal Energy Science, Shanghai Key Laboratory of Special Artificial Microstructure Materials and Technology, School of Physics Science and Engineering, Tongji University, Shanghai 200092, China; 2 Dipartimento di Fisica, Università di Camerino, I-62032 Camerino, Italy; 3 μFlow Group, Department of Chemical Engineering, Vrije Universiteit Brussel, 1050 Brussels, Belgium; 4 Department of Chemistry, Presidency University, Kolkata 700073, India

**Keywords:** active matter, stochastic resetting, Brownian motion, diffusion

## Abstract

We propose a two-dimensional model of biochemical activation process, whereby self-propelling particles of finite correlation times are injected at the center of a circular cavity with constant rate equal to the inverse of their lifetime; activation is triggered when one such particle hits a receptor on the cavity boundary, modeled as a narrow pore. We numerically investigated this process by computing the particle mean-first exit times through the cavity pore as a function of the correlation and injection time constants. Due to the breach of the circular symmetry associated with the positioning of the receptor, the exit times may depend on the orientation of the self-propelling velocity at injection. Stochastic resetting appears to favor activation for large particle correlation times, where most of the underlying diffusion process occurs at the cavity boundary.

## 1. Introduction

The goal of the narrow escape problem in diffusion theory [1] is to calculate the mean-first exit time (MFET) of a massless (overdamped) Brownian particle through a small absorbing area (hole or pore) on the otherwise reflecting boundary of a closed cavity. The renewed interest for this problem, which dates back to Lord Rayleigh, is due to its relevance in molecular biology and biophysics. Small holes have been often invoked to model localized targets on a cellular membrane, such as protein pores, receptors for neurotransmitter molecules in neuronal synapses, or narrow necks in neuronal spines [1,2]. From a mathematical viewpoint, the narrow escape problem is equivalent to calculating the principal eigenvalue of the mixed Dirichlet–Neumann problem for the Laplace equation in a bounded domain, whose Dirichlet boundary is only a small patch on the otherwise Neumann boundary [2]. Indeed, the principal eigenvalue approximates asymptotically the reciprocal of the MFET in the limit of shrinking patch size [2]. Further applications of this problem can be found in microfluid technology [3] and transport theory at large [4].

**Figure 1:**
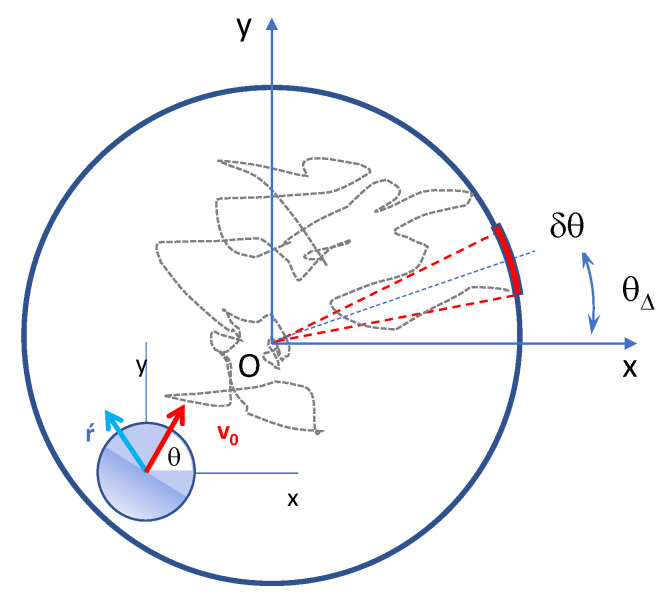
Scheme of the 2D biochemical activation process: diffusing particle injected at the center, *O*, of a circular cavity; chemoreceptor modeled by an absorbing arc of angular width δθ and oriented at an angle $\theta_{\Delta }$ with respect to the horizontal axis. The injected particle is a JP with fixed self-propelling speed, $v_{0}$, and other dynamical parameters as sketched in the inset (see Equation (1)).

To fix notation, we consider for simplicity the idealized two-dimensional (2D) model of biochemical activation illustrated in Figure 1. Brownian particles are injected at the center of a circular cavity and freely diffuse up to the cavity boundary, which is reflecting everywhere except for a small patch of angular size, δθ≪2π, representing an absorbing chemoreceptor. As customary in most biophysical models, the particles are assumed massless, to neglect the spatial correlations due to inertia, and pointlike, to minimize unavoidable hydrodynamic effects in the vicinity of the pore [5]. Activation occurs when a particle is absorbed by the chemoreceptor. As long as the injected particle undergoes standard Brownian motion with diffusion constant $D_{0}$, its MFET (the activation time) can be well approximated by employing the refined analytical techniques developed over the last two decades by the authors of Ref. [2]. Their systematic approach has been validated by many numerical studies [2,4].

Two recent developments in the theory of classical diffusion suggest readdressing this otherwise well-understood problem, namely, the notions of active Brownian motion and stochastic resetting. The most tractable example of active Brownian motion is represented by microswimmers, namely tiny Brownian particles, either biological or synthetic, capable of self-propulsion in an active medium [6,7]. A class of artificial swimmers widely investigated in the current literature are the so-called Janus particles (JPs), mostly spherical colloidal particles with two differently coated hemispheres, or “faces” [8,9]. Thanks to their functional asymmetry, JPs are capable of harvesting environmental energy and convert it into kinetic energy. Therefore, active particles are modeled to be autonomously propelling with constant speed, $v_{0}$, and orientation fluctuating in time with constant rate, $D_{\theta }$. Their Brownian motion is thus characterized by a finite persistence time, $\tau_{\theta }=1/D_{\theta }$, even in the absence of inertia effects [10]. Numerical studies of the narrow escape problem for active particles [11,12,13,14,15] confirm that effects due to their spatial correlation dominate when the corresponding persistence length, $l_{\theta }=v_{0}/D_{\theta }$, is of the order of or larger than the cavity size, as anticipated in Ref. [16]. In the following, we consider for convenience a spherical JP as an archetypal model of self-propelling Brownian particle, though our conclusions can be easily extended to any kind of microswimmer, biological and artificial alike.

First passage properties under resetting is another topic of current interest in the field of stochastic processes (see Ref. [17] for a recent review). Stochastic resetting (SR) refers to the sudden interruption at random times of a stochastic process followed by its starting anew, possibly after a latency time, with the same initial conditions. Diffusion under SR is a nonequilibrium stationary process, which found applications in search problems [18], optimization of randomized computer algorithms [19], and in many biophysical problems [20,21]. Surprisingly, for a freely diffusive Brownian particle under SR, the otherwise infinite mean first passage time from the injection point to an assigned target point, becomes finite, and, most notably, can be minimized for an optimal value of the resetting time, τ [22,23]. Many analytical methods earlier developed in the theory of homogeneous stochastic processes can be generalized to study diffusion under SR, for instance, to calculate the MFET of a reset particle out of a one-dimensional domain [24]. To the best of our knowledge, the narrow escape problem under SR has never been addressed in the literature. A closely related study is Ref. [25], wherein the 2D diffusion of a stochastically reset free-Brownian particle is investigated analytically: SR is shown to critically slow down diffusion, with strongly anomalous means-square displacement proportional to $\ln^{2}\left(\tau t\right)$, without totally suppressing it.

By combining the above diffusion mechanisms, two further ingredients of our biochemical activation model come into play, namely (i) self-propelling injected particles to mimic activation by motile objects, like biomolecules or cells and (ii) particles of finite lifetime, τ, injected with rate 1/τ. This corresponds to the assumption that the injection mechanism works under SR conditions with time constant τ and zero latency time. By means of extensive numerical simulations, we show how both model extensions affect the relevant activation times, i.e., the particle MFET’s through the narrow hole representing the chemoreceptor.

The contents of the present paper is organized as follows. In Section 2 we detail the dynamics of an active JP of fixed speed, $v_{0}$, and angular persistence time, $\tau_{\theta }$, confined to a circular cavity of radius $r_{0}$. Reflecting-sliding boundary conditions (b.c.) are assumed except for a short absorbing arc of angular size δθ. In Section 3 we discuss the dependence of the MFET of an active JP on the noise strengths, $D_{0}$ and $D_{\theta }$, and the opening size, δθ. More importantly, as the presence of an absorbing path on the boundary breaks the circular symmetry of the problem, we consider two different trajectory averages, namely, injection angles, $\theta_{0}=\theta \left(0\right)$, uniformly distributed in the interval [0,2π], or pointing to the escape hole, $\theta_{0}=\theta_{\Delta }$ (Figure 1). In Section 4 we analyze the escape problems of Section 3 under SR to determine under what circumstances resetting favors the activation process. Finally, in Section 5 we discuss possible extensions of the present work in view of technological applications.

## 2. Model

As anticipated in Section 1, to avoid inessential technicalities, we restricted our investigation to the case of a 2D circular cavity of radius $r_{0}$ and to pointlike artificial microswimmers of the JP class. The extension of our conclusions to 3D cavities of different geometries is straightforward [2,26]. An active JP gets a continuous push from the suspension fluid, which in the overdamped regime amounts to a self-propulsion velocity of constant modulus, $v_{0}$, and fluctuating orientation, θ, measured with respect to the *x*-axis in Figure 1. The bulk dynamics of such JP obeys the Langevin equations (LE) [16,27]

(1) $$\begin{array}{ccc} \dot{x} & = & v_{0}\cos\theta +\xi_{x}\left(t\right)\\ \dot{y} & = & v_{0}\sin\theta +\xi_{y}\left(t\right)\\ \dot{\theta } & = & \xi_{\theta }\left(t\right), \end{array}$$

where r=(x,y) are the coordinates of the particle center of mass subject to the Gaussian noises $\xi_{i}\left(t\right)$, with $\langle \xi_{i}\left(t\right)\rangle =0$ and $\langle \xi_{i}\left(t\right)\xi_{j}\left(0\right)\rangle =2D_{0}\delta_{ij}\delta \left(t\right)$ for i=x,y, modeling the equilibrium thermal fluctuations in the suspension fluid. The orientational fluctuations of the propulsion velocity are modeled by the Gaussian noise $\xi_{\theta }\left(t\right)$ with $\langle \xi_{\theta }\left(t\right)\rangle =0$ and $\langle \xi_{\theta }\left(t\right)\xi_{\theta }\left(0\right)\rangle =2D_{\theta }\delta \left(t\right)$, where $\tau_{\theta }=1/D_{\theta }$ turns out to be the relaxation (or persistence) time of the self-propulsion velocity [27]. All noise sources in Equation (1) have been treated as independently tunable, although, strictly speaking, thermal and orientational fluctuations may be statistically correlated. Moreover, by considering pointlike particles, we ignored hydrodynamic effects, like their capture by the cavity walls [28]. However, we made sure that the remaining parameters used in our simulations are experimentally accessible, as apparent by expressing times in seconds and lengths in microns [13,16]. We remind that the process r(t) in Equation (1) is non-Gaussian, but its mean-square displacement (MSD) approaches asymptotically, for $t\gg \tau_{\theta }$, the normal diffusion law, $\langle r{\left(t\right)}^{2}\rangle -{\langle r\left(t\right)\rangle }^{2}=4Dt$ with [27]

(2) $$\begin{array}{c} D=D_{0}+v_{0}^{2}/2D_{\theta }. \end{array}$$

Simulating a JP confined into a circular cavity of radius $r_{0}$, $x^{2}+y^{2}\leq r_{0}^{2}$, requires defining its collisional dynamics at the boundary. For the translational velocity, $\vec{\dot{r}}$, we assumed standard boundary conditions [1]. Regarding the angular coordinate, the simplest choice is represented by sliding b.c.: the self-propulsion vector, ${\vec{v}}_{0}=v_{0}\left(\cos\theta ,\sin\theta \right)$, keeps pointing in the same direction, θ, until the noise $\xi_{\theta }\left(t\right)$ redirects it toward the interior of the cavity. As a result, the particle tends to slide along the cavity wall. Absorbing b.c. are imposed on the arc of angular width δθ representing the cavity hole: as the particle touches it, the simulation run is stopped and the relevant exit time, *T*, recorded. The MFET is computed as the average, 〈T〉, taken over a large number of trajectories (typically 10${}^{5}$). The extension to randomized b.c. is also possible [16], but at the price of unnecessary complications.

The LE (1) could be conveniently reformulated in dimensionless units by rescaling $x\to x/r_{0}$$y\to y/r_{0}$, and $t\to (v_{0}/r_{0})t$. The remaining independent parameters would be then rescaled as $D_{0}\to D_{0}/r_{0}v_{0}$ and $D_{\theta }\to \left(r_{0}/v_{0}\right)D_{\theta }$. Without loss of generality, one can set $r_{0}=1$ and $v_{0}=1$ and the ensuing simulation results can be regarded as expressed in dimensionless units. The stochastic differential Equation (1) were numerically integrated by means of a standard Milstein scheme [29]. Particular caution was exerted in the low-noise regime, i.e., for vanishingly small $D_{0}$ and $D_{\theta }$, because transients can grow exceedingly long, thus affecting the MFET computation.

So far we have made no mention of SR. The resetting protocols will be introduced more conveniently at a later point in Section 4. In Table 1, for reader’s convenience, we list all model parameters and relevant symbols.

## 3. Narrow Escape of Active Particles

**Figure 2:**
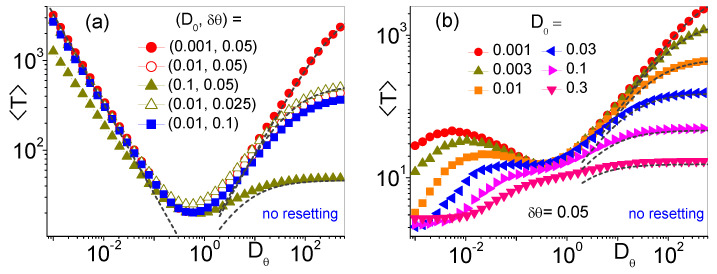
〈T〉 vs. $D_{\theta }$ for different $D_{0}$ and δθ (see legends). Other simulation parameters are: $r_{0}=1$, $v_{0}=1$ and averages were taken over 10${}^{5}$ trajectories with initial orientation, $\theta_{0}$, of the self-propelling JP either uniformly distributed (**a**) or pointing toward the center of the absorbing hole, $\theta_{0}=\theta_{\Delta }$ (**b**). Dashed curves on the right (left) represent the analytical predictions for large (small) $D_{\theta }$ in Equation (3) [Equation (4)].

We consider first the MFET for a self-propelling microswimmer, like an active JP, in the absence of SR. This is a natural extension of the models discussed in Ref. [2], whereby the passive diffusing Brownian particle is replaced by an active one. The averages of the exit times will be taken under two different i.c. for $\theta_{0}=\theta \left(0\right)$, as illustrated in Figure 2: (i) completely random, as is the case of injected microswimmers, on the orientation of which we have no control; (ii) directed on target, as is the case of artificial microrobots one can suitably orient prior to injection [30].

*(i) Uniform $\theta_{0}$ distribution*. A detailed analysis of this case is reported in Ref. [13]. We summarize here a few key results we will recall in the following sections. In the limit of narrow pores, δθ≪2π, and vanishingly small translational noise, $D_{0}\to 0$, the curves of 〈T〉 versus $D_{\theta }$, $\langle T\left(D_{\theta }\right)\rangle$, exhibit two distinct regimes. For $l_{\theta }\ll r_{0}$, i.e., short persistence times, the JP behaves like a regular Brownian particle. Indeed, its MFET is expected to be much larger than $\tau_{\theta }$, so that the analytical estimate derived in Ref. [2] holds, namely,

(3) $$\begin{array}{c} \langle T\left(D_{\theta }\right)\rangle =\frac{r_{0}^{2}}{D}\left[\ln\left(\frac{4}{\delta \theta }\right)+\frac{1}{4}\right]. \end{array}$$

The escape process in Equation (3) is governed by the effective, $D_{\theta }$-dependent translational diffusion constant, *D*, introduced in Equation (2). The analytical prediction of Equation (3) fits quite closely the rising branches of the $\langle T\left(D_{\theta }\right)\rangle$ curves in Figure 2a.

Conversely, for $l_{\theta }\gg r_{0}$, i.e., long persistence times, the MFETs are apparently proportional to $\tau_{\theta }$. This signals that the exit process is governed by the particle diffusion along the cavity boundary. On a time scale of the order of $r_{0}/v_{0}$, the JP reaches the boundary and then slides along it, until its self-propulsion vector $v_{0}$ is oriented parallel to r=(x,y). The ensuing slow diffusion parallel to the cavity wall is driven by the angular fluctuations, see third LE (1). The mean-first passage time for the particle to diffuse along the cavity wall up to the absorbing hole can be calculated analytically [1,4]. For δθ≪2π one obtains [13]

(4) $$\begin{array}{c} \langle T\left(D_{\theta }\right)\rangle =\pi^{2}/3D_{\theta }, \end{array}$$

also in good agreement with the numerical data displayed in Figure 2a. Deviations from the large-$\tau_{\theta }$ estimate of 〈T〉 occur in the presence of appreciable translational fluctuations. During the time, $r_{0}/v_{0}$, it takes to reach the cavity wall, the particle undergoes angular MSD, $\Delta^{2}\theta =\langle {\left(\theta \left(t\right)-\theta_{0}\right)}^{2}\rangle$, due to two separate fluctuation sources: (1) the angular noise itself, $\xi_{\theta }\left(t\right)$ of the third LE (1), causing an angular MSD of the particle orientation, $\Delta^{2}\theta_{\mathrm{ang}}=2D_{\theta }\left(r_{0}/v_{0}\right)$, and (2) the translational noises, $\xi_{x}\left(t\right)$ and $\xi_{y}\left(t\right)$ of the first two LE’s (1), responsible for an additional angular dispersion of the particle along the cavity wall, $\Delta^{2}\theta_{\mathrm{trans}}=2\left(D_{0}/r_{0}^{2}\right)\left(r_{0}/v_{0}\right)=2D_{0}/r_{0}v_{0}$. We recall that the angular and translational noises have been assumed to be statistically independent. On comparing $\Delta \theta_{\mathrm{ang}}$ and $\Delta \theta_{\mathrm{trans}}$, one concludes that the derivation of Equation (4) holds good for

(5) $$\begin{array}{c} D_{0}≲r_{0}^{2}D_{\theta }, \end{array}$$

when the role of the translational noises is negligible.

The crossover between the short and large $\tau_{\theta }$ regimes, i.e., between translational and angular diffusion regimes, is marked by a dip in the $\langle T\left(D_{\theta }\right)\rangle$ curves, not much sensitive to the parameters δθ and $D_{0}$. Indeed, the position of the $\langle T\left(D_{\theta }\right)\rangle$ dips can be estimated by equating the r.h.s. of Equations (3) and (4), that is,

(6) $$\begin{array}{c} \frac{r_{0}}{v_{0}}D_{\theta }^{\min}≃\frac{\pi /\sqrt{6}}{\ln^{1/2}\left(4/\delta \theta \right)}. \end{array}$$

Here we have made use of the fact that, upon decreasing $D_{\theta }$ at small $D_{0}$, the effective diffusion constant of Equation (2) grows independent of $D_{0}$, i.e., $D\to v_{0}^{2}/2D_{\theta }$. The question remains of whether such MFET dips signal the onset of any new mechanism in the escape process. This question will be addressed in Section 4.

*(ii) Injected particle aimed at target, $\theta_{0}=\theta_{\Delta }$*. At variance with the current literature [11,12,13], we postulate now that the diffusing JP is injected with self-propulsion vector, $v_{0}$, pointing toward the exit pore. This implies that the averages 〈T〉 must be taken over samples of statistically independent trajectories with fixed initial angle, $\theta_{0}=\theta_{\Delta }$. A few examples of such averages are displayed in Figure 2b. Like in *(i)*, short and large $\tau_{\theta }$ regimes are clearly distinguishable, respectively for $D_{\theta }≳D_{\theta }^{\min}$ and $D_{\theta }≲D_{\theta }^{\min}$. In the former, that is for $l_{\theta }\ll r_{0}$, the averaging protocol is seemingly irrelevant. Indeed, being the expected MFET much larger than $\tau_{\theta }$, memory of the i.c., $\theta_{0}=\theta \left(0\right)$, is lost well before the particle approaches the cavity wall. It is no surprise that the corresponding curves $\langle T\left(D_{\theta }\right)\rangle$ of Figure 2a,b coincide for $D_{\theta }≳D_{\theta }^{\min}$.

**Figure 3:**
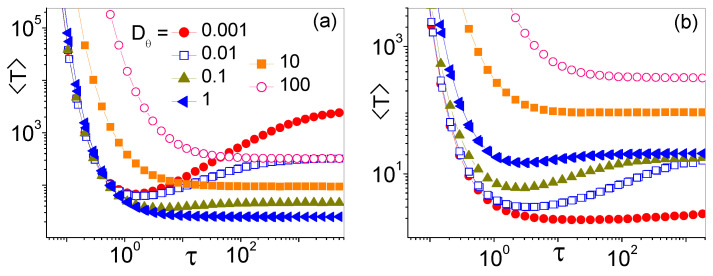
〈T〉 vs. τ for different $D_{\theta }$, see legends. Other simulation parameters are: δθ=0.05, $r_{0}=1$, $v_{0}=1$, and $D_{0}=0.01$. Averages were taken over 10${}^{5}$ trajectories with initial orientation either uniformly distributed (**a**) or pointing toward the center of the absorbing hole, (**b**).

In stark contrast, for $D_{\theta }≲D_{\theta }^{\min}$, the choice of the initial particle orientation plays a prominent role. Upon decreasing $D_{\theta }$ below $D_{\theta }^{\min}$, the $\langle T\left(D_{\theta }\right)\rangle$ curves of Figure 2b turn first upward, because, like in Figure 2a, for $l_{\theta }>r_{0}$ the escape process is still governed by the angular diffusion against the cavity wall. However, in the limit $D_{\theta }\to 0$, all the MFET curves of Figure 2b appear to bend toward a lower horizontal asymptote. A simple estimate of the asymptotic value 〈T(0)〉 is obtained by noticing that for $D_{\theta }\to 0$ the self-propulsion vector $v_{0}$ of the injected JP points straight to the exit hole and is unlikely to change direction before hitting it; hence in ballistic approximation,

(7) $$\begin{array}{c} \langle T\left(0\right)\rangle ≃r_{0}/v_{0}. \end{array}$$

(See also Figure 3b in the regime of large SR time.) The crossover between the ballistic regime of Equation (7) and the angular diffusion regime of Equation (4) is rather smooth. Broad MFET peaks emerge only for small values of $D_{0}$ and shift toward lower $D_{\theta }$ with decreasing $D_{0}$. As discussed in *(i)*, this crossover is a consequence of the competition between angular and translational diffusion against the cavity wall: the angular diffusion regime of Equation (4) fails in the presence of translational noises, when $\Delta^{2}\theta_{\mathrm{trans}}≳\Delta^{2}\theta_{\mathrm{ang}}$ i.e., for $D_{0}≳r_{0}^{2}D_{\theta }$ (compare with Equation (5)). The interplay of translational and angular noises was hardly detectable in the curves of Figure 2a.

## 4. Narrow Escape under Stochastic Resetting

We address now the narrow escape problem of Section 2 under SR. We assume that the diffusing particle has a random lifetime, $t_{i}$, with exponential distribution, $p\left(t_{i}\right)=e^{-t_{i}/\tau }/\tau$. A time $t_{i}$ after injection, the diffusing particle is replaced with an identical one, injected at the center of the cavity, possibly after a latency time. This mechanism has been invoked, for instance, to develop random optimization algorithms, to address foraging and other search problems, and to model polymer growth [2]. Following the discussion of Section 3, its self-propulsion velocity, ${\vec{v}}_{0}$, can be initially oriented either uniformly, or toward the absorbing pore representing the chemoreceptor, $\theta_{0}=\theta_{\Delta }$. For simplicity, the replacement of a diffusing particle with a new injected one occurs instantaneously, that is with zero latency time. The activation time, *T*, is then defined as the time elapsed between two escapes, no matter how many stochastic resettings took place in between.

The dependence of the MFET on the SR time-constant, τ, for different values of the persistence time, $\tau_{\theta }$, is illustrated in Figure 3a,b, respectively, for uniformly distributed $\theta_{0}$ and $\theta_{0}=\theta_{\Delta }$. In Figure 3a all curves 〈T(τ)〉 for $D_{\theta }>D_{\theta }^{\min}$ decay monotonically with τ, to the corresponding $\langle T\left(D_{\theta }\right)\rangle$ in the absence of SR, see Figure 2a. Vice versa, for $D_{\theta }<D_{\theta }^{\min}$ the curves 〈T(τ)〉 go through a minimum for an optimal SR time, $\tau_{SR}$, and, remarkably, diverge for τ→0, insensitive to the persistence time, $\tau_{\theta }$. We notice that these properties hold only at low translational noise levels, that is under the condition in Equation (5).

The picture of Figure 3b looks more complicated. In contrast with Figure 3a, the profile of the curves 〈T(τ)〉 turns monotonic for vanishingly small values of $D_{\theta }$. This happens in coincidence with the onset of the ballistic regime of Equation (7) in Figure 2b. The first conclusion we draw is that SR favors the narrow escape process of active JP’s for both averaging protocols, but only in the angular diffusion regime of Equation (5). On the contrary, in the ballistic regime of Equation (7) (for $\theta_{0}=\theta_{\Delta }$) and in the translational diffusion regime of Equation (3), SR slows the activation process down. These remarks also answer our question about the meaning of the dips in the $\langle T\left(D_{\theta }\right)\rangle$ curves of Figure 2: Those dips mark a relatively smooth transition from the translational to the angular diffusion regimes, transition that appears there more prominently due to the $D_{\theta }$ dependence of the effective diffusion constant *D* in Equation (2).

**Figure 4:**
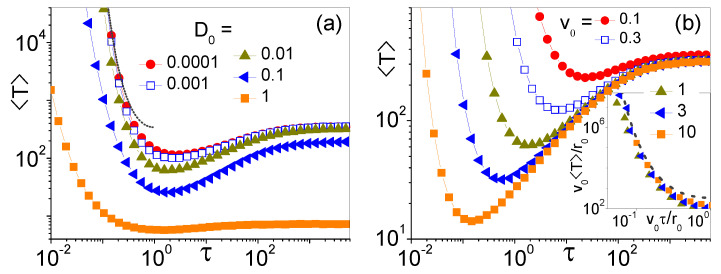
〈T〉 vs. τ for different (**a**) $D_{0}$ and (**b**) $v_{0}$ (see legends). Other simulation parameters are: $r_{0}=1$, δθ=0.05, $D_{\theta }=0.01$ and (**a**) $v_{0}=1$, (**b**) $D_{0}=0.01$. All averages were taken over 10${}^{5}$ trajectories with uniformly distributed initial orientation, $\theta_{0}$. In the inset of panel (**b**) are the rescaled data (with *T* and τ in units of $r_{0}/v_{0}$) from the main panel with $v_{0}\geq 1$. Dashed curves in (**a**) and the inset of (**b**) represent the analytical estimates of Equation (8).

The τ-dependence of the MFET’s under SR tells us something new about the statistics of the narrow escape process. As discussed in Ref. [21], the monotonic decay of 〈T(τ)〉 implies that the escape time, *T*, is a point random process with small volatility, i.e., $\left(\langle T^{2}\rangle -{\langle T\rangle }^{2}\right)/{\langle T\rangle }^{2}\leq 1$. However, this is not the case of escape processes in the angular diffusion regime, where the MFET is shortened by the SR protocol. The minima of the 〈T(τ)〉 curves plotted in Figure 4 occur for optimal SR times, $\tau_{SR}≃r_{0}/v_{0}$, apparently independent of $D_{\theta }$ and $D_{0}$. This is consistent with the conditions $D_{\theta }≲D_{\theta }^{\min}$ and $D_{0}≲r_{0}^{2}D_{\theta }$ (see Equations (6) and (5), which define the angular diffusion regime of Equation (4)). The latter points to the irrelevance of the translational noise level, while the former can be recast as $\tau_{\theta }≲\tau_{SR}$.

Under the same conditions, for $\tau \ll \tau_{SR}$ the 〈T(τ)〉 curves of Figure 4 diverge independently of $D_{\theta }$ and $D_{0}$. This is in agreement with the analysis of Ref. [31]. Indeed, for vanishingly small τ, the MFET is dominated by the particle diffusion from the center to the cavity wall. As the self-propulsion vector, $v_{0}$, acts here like an effective constant force pointing outward, we apply Equation (6) of Ref. [31], to determine the mean-first passage time the particle takes to reach the cavity boundary, namely, for $v_{0}^{2}\gg 4D/\tau$, $\tau e^{r_{0}/\left(v_{0}\tau \right)}$. However, on neglecting the slow angular diffusion along the boundary, the probability that in this time interval the particle gets absorbed by the chemoreceptor is proportional to its angular size, δθ/2π. This leads to our estimate for the diverging branches of 〈T(τ)〉,

(8) $$\begin{array}{c} \langle T\left(\tau \right)\rangle =\tau \left(2\pi /\delta \theta \right)e^{r_{0}/\left(v_{0}\tau \right)}, \end{array}$$

drawn in Figure 4a and, after rescaling, in the inset of Figure 4b. In dimensionless time units, all diverging branches collapse on a universal curve.

Note that in the opposite limit of $v_{0}^{2}\ll 4D/\tau$ (not shown here), we would obtain $\langle T\left(\tau \right)\rangle =\tau \left(2\pi /\delta \theta \right)e^{r_{0}/\sqrt{D_{0}\tau }}$, consistently with Equation (15) of Ref. [24] for a passive tracer.

In both self-propulsion limits the MFET diverges exponentially for τ→0. This reflects the strong suppression of the 2D diffusion mechanism caused by SR, as detailed in Ref. [25] for $v_{0}=0$. Stated otherwise, particle confinement is due to the cavity walls and narrow crossing occurs for any choice of our model parameters.

## 5. Conclusions

In this paper, we have investigated a study model of biochemical activation, whereby an activating particle is injected at the center of a circular cavity and taken out through a small opening, representing a chemoreceptor. The activation mechanism is completed after the injected particle has diffused up to the absorbing opening. We focused on two new aspects of this otherwise well-established problem: (i) the injected particle has finite persistence time, $\tau_{\theta }$, related to its self-propulsion mechanism (active Brownian motion), and (ii) in the case of particles with finite lifetime, τ, we assume that the activating particles are continuously injected with constant rate 1/τ (stochastic resetting). By extensive numerical simulations, we have determined the dependence of the activation time, i.e., the injected particle MFET, on both time scales, $\tau_{\theta }$ and τ.

In view of technological applications, we remark that this model can be easily generalized to account for two more ingredients. First, a finite persistence time is not a unique signature of active Brownian motion. Indeed, any Brownian particle of mass *m*, in thermodynamical equilibrium at temperature *T*, has finite correlation time $\tau_{\beta }=m/\beta$, with β denoting its viscous constant. When the corresponding correlation length, $v_{\mathrm{th}}\tau_{\beta }$, with $v_{\mathrm{th}}=\sqrt{kT/m}$, grows comparable to the cavity size, a nonmonotonic dependence of the particle’s MFET is also to be expected.

Secondly, we assumed that particles failing to hit the assigned target within their finite lifetime, τ, were instantaneously reinjected at the center of the cavity. In this way, we made sure that the SR time constant coincided with the particle lifetime. We note, however, that this is not a necessary condition for our model to work. One can easily extend it to the case when τ is shorter than the SR time constant, thus allowing for a nonzero latency time between particle removal and reinjection [20]. The only restriction one needs to impose is that the cavity contains only one diffusing particle at a time.

## Figures and Tables

**Table 1:** Model parameters.

Symbol	Parameter	Remarks
$D_{0}$, *D*	free, effective diffusion constant	*D* defined in Equation (2)
$D_{\theta }$	angular diffusion constant	tunable
$l_{\theta }$	self-propulsion length	$l_{\theta }=v_{0}\tau_{\theta }$
$r_{0}$	cavity radius	$r_{0}=1$
〈T〉	MFET through pore	
$v_{0}$	self-propulsion speed	$v_{0}=1$
$\theta_{\Delta }$, δθ	pore position, width	δθ≪2π
τ	average SR time	$\tau_{SR}$ optimal SR time
$\tau_{\theta }$	angular relaxation time	$\tau_{\theta }=1/D_{\theta }$

## Data Availability

Data can be available upon reasonable request from all the authors.

## Abbreviations

The following abbreviations are used in this manuscript:

MFET	mean-first exit time
SR	stochastic resetting
